# ^223^Ra-Dichloride in castration-resistant metastatic prostate cancer: improving outcomes and identifying predictors of survival in clinical practice

**DOI:** 10.1007/s00259-018-4083-3

**Published:** 2018-07-11

**Authors:** Sabina Dizdarevic, Maryam Jessop, Patrick Begley, Sean Main, Angus Robinson

**Affiliations:** 1 Brighton and Sussex University Hospitals NHS Trust, Brighton, UK; 2 Department of Nuclear Medicine, Royal Sussex County Hospital, Eastern Road, Brighton, BN2 5BE UK; 3 Brighton and Sussex Medical School, Brighton, UK

**Keywords:** Alkaline phosphatase, Bone metastases, Metastatic castration-resistant prostate cancer, ^223^Ra-Dichloride, Referral patterns, Radium

## Abstract

**Purpose:**

We first assessed whether the pattern of referrals to a nuclear medicine clinic improved as experience with ^223^Ra-dichloride increased, and whether referral patterns affected patient outcomes, and second assessed the value of bone scintigraphy, total alkaline phosphatase (tALP) and lymphadenopathy as prognostic factors in patients receiving ^223^Ra-dichloride.

**Methods:**

A total of 57 patients eligible to receive ^223^Ra-dichloride over a 2-year period (March 2014 to March 2016) were retrospectively assessed and prospectively followed (median follow up 298 days). ^223^Ra-Dichloride was administered at 4-week intervals for a maximum of six injections. The numbers of patients in years 1 and 2 referred in relation to extent of bone disease (EOBD) category and overall survival (OS) were determined. The prognostic factors EOBD category, baseline tALP (tALP_BL_), tALP response, greatest percentage reduction in tALP from baseline in any treatment cycle (ALP_max_; among patients with elevated ALP_BL_), and the presence of lymphadenopathy were assessed as predictors of OS.

**Results:**

The proportion of patients with EOBD1 was higher in year 2 than in year 1 (29% and 4%, respectively), and in year 2 there was a lower rate of symptomatic skeleton-related events, a higher proportion of patients completing six cycles, and longer (albeit nonsignificant) OS (*p* = 0.55). There were significant differences in OS between EOBD4 patients and those in all other groups and between EOBD1 and EOBD3 patients (*p* < 0.05). OS was longer in patients with normal tALP_BL_ than in those with elevated tALP_BL_ (*p* = 0.01), in ALP responders than in nonresponders (*p* < 0.05), and in patients without lymphadenopathy than in those with lymphadenopathy (*p* = 0.29). OS was correlated with ALP_max_ (*r*^2^ = 0.24).

**Conclusion:**

A collaborative multidisciplinary referrals pathway, together with increased experience with ^223^Ra-dichloride, led to improved outcomes. In patients with elevated tALP_BL_, tALP dynamics may be useful for monitoring response and predicting OS. Imaging and prognostic markers may therefore be of value for individualizing ^223^Ra-dichloride treatment and planning retreatment; however, further studies are required.

## Introduction

Castration-resistant prostate cancer (CRPC) is defined as disease progression with rising serum prostate-specific antigen (PSA) levels or radiological progression despite documented castrate levels of testosterone [1]. While 4% of newly diagnosed prostate cancer patients under the age of 75 years present with metastases [2], 85% of patients have proven evidence of metastases on diagnosis of castration resistance [3]. Following the development of CRPC, the incidence of metastatic disease and/or radiotherapy (a surrogate for disease progression) has been reported to be 6.4 per 100 person-years [4].

As there is no cure for metastatic CRPC (mCRPC), the goal of treatment is to prolong life and improve quality of life (QoL) [5]. According to the National Comprehensive Cancer Network (NCCN) and the American Society of Clinical Oncology (ASCO) guidelines, treatment options for mCRPC include: sipuleucel-T (for asymptomatic or minimally symptomatic mCRPC; available in the USA only), androgen-targeted therapies including abiraterone with prednisone or enzalutamide (for asymptomatic or minimally symptomatic mCRPC), cytotoxic chemotherapies including docetaxel or cabazitaxel (for symptomatic mCRPC, often but not exclusively with visceral metastases), ^223^Ra-dichloride (for symptomatic mCRPC with bone metastases and without visceral metastases), and zoledronic acid or denosumab (for patients with bone metastases to prevent skeleton-related events). Continued concurrent androgen-deprivation therapy (ADT) is also recommended [5, 6]. Treatment recommendations are dependent on tumour burden, prior therapy, whether the patient is asymptomatic, minimally symptomatic or symptomatic, the presence of visceral or bone metastases, and Eastern Cooperative Oncology Group (ECOG) performance status [5, 6]. In the UK, the 2014 National Institute for Health and Care Excellence (NICE) guidelines recommend docetaxel (for patients with a Karnofsky performance status of ≥60%), corticosteroids such as dexamethasone (if ADT and antiandrogen therapy have failed), and bone-targeted therapies including bisphosphonates (for pain relief when other treatments have failed), strontium-89 (in patients with painful bone metastases) [7] and, more recently (2016), ^223^Ra-dichloride (in patients with mCRPC, symptomatic bone metastasis and no evidence of visceral metastasis [8]).

^223^Ra-Dichloride is a targeted alpha-emitter therapy that mimics calcium and targets areas of increased bone turnover. Alpha particles have a very short range (<100 μm), so damage to surrounding tissues is minimal [9]. Prior to the introduction of ^223^Ra-dichloride, bone-targeting therapies did not lead to longer overall survival (OS); the benefits were primarily limited to pain relief and delay of skeletal events [10]. ^223^Ra-Dichloride is approved in the UK for the ‘treatment of adults with CRPC, symptomatic bone metastases and no known visceral metastases’ [8, 11]. The ALSYMPCA study investigated the efficacy of ^223^Ra-dichloride in 921 patients with mCRPC and symptomatic bony metastases [12]. The primary endpoint was OS; secondary efficacy endpoints included time to the first symptomatic skeletal event and various safety and QoL endpoints. Overall, ^223^Ra-dichloride showed benefits in terms of OS and QoL compared with placebo. Despite these very positive results, to date, few reliable prognostic factors have been identified in mCRPC patients undergoing treatment with ^223^Ra-dichloride [13].

The purpose of the current study was to describe real-world experience with ^223^Ra-dichloride at a single centre in the UK (Brighton and Sussex University Hospital), and to assess bone scintigraphy, a biochemical biomarker (total alkaline phosphatase, tALP) and coexisting lymph node disease as prognostic factors. The study also aimed to assess whether the pattern of referrals to the nuclear medicine clinic improved as experience with ^223^Ra-dichloride increased, and to evaluate whether referral patterns influenced patient outcomes.

## Materials and methods

### Study design

This study was a retrospective and prospective review of all patients who were referred to the Nuclear Medicine Department of Brighton and Sussex University Hospital over a 2-year period (March 2014 to March 2016) who were eligible to receive treatment with ^223^Ra-dichloride. These patients were defined as those with mCRPC, symptomatic bone metastases and no known visceral metastatic disease. Patient records were reviewed using patient notes, ChemoCare electronic records (e.g. treatment schedule), the picture and archiving system (imaging), and laboratory blood test data. All patients gave written informed consent for their diagnostic and therapeutic management and follow up.

### Treatment schedule

^223^Ra-Dichloride was administered via intravenous injection at 4-week intervals for a maximum of six injections (in accordance with prescribing information). The administered volume was calculated as described in the prescribing information [11]:

$$ \frac{\mathrm{Patient}\ \mathrm{body}\ \mathrm{weight}\ \left(\mathrm{kg}\right)\ \mathrm{x}\ \mathrm{dose}\ \left(55\ \mathrm{kilobecquerel}/\mathrm{kg}\ \mathrm{body}\ \mathrm{weight}\right)}{\mathrm{Decay}\ \mathrm{correction}\ \mathrm{factor}\ \mathrm{x}\ 1100\ \mathrm{kilobecquerel}/\mathrm{mL}} $$

Patients who developed visceral metastases, as identified by computed tomography (CT) prompted by a sudden change in clinical presentation and a marked increase in biochemical markers (tALP and/or PSA), were required to discontinue treatment.

### Referral patterns

Various practical strategies were put in place at the institution to improve clinician-to-patient communication and establish a more coherent interface between uro-oncology and nuclear medicine, with a view to optimizing the patient pathway and improving patient outcomes. These strategies have been discussed in more detail elsewhere [14]. Improvements in referral patterns were assessed by comparing the proportions of patients referred to the Nuclear Medicine Department in year 1 and year 2 for each extent of bone disease (EOBD) category. EOBD was assessed using bone scintigraphy, and the categories were as follows: *1* ≥2 but <6 metastases, *2* 6–20 metastases, *3* >20 metastases but less than a superscan, *4* superscan (diffuse involvement i.e. more than 75% of the ribs, vertebrae and pelvic bones) [12, 15].

### Effect of referral patterns on outcomes

OS, defined as the time from first ^223^Ra-dichloride treatment in the nuclear medicine clinic to the date of death from any cause, was assessed in the year-1 and year-2 patients. The incidence of symptomatic skeleton-related events (SSREs) was also determined. An SSRE was defined as any skeleton-related adverse event or any radiotherapy to bone.

### Prognostic factors

#### EOBD category

OS and number of completed treatment cycles were recorded according to EOBD status.

#### Alkaline phosphatase

Total ALP (tALP) was assessed at baseline (i.e. before treatment; ALP_BL_) and before every subsequent cycle. Normal ALP_BL_ was defined as ≤130 U/L, and elevated ALP_BL_ was defined as >130 U/L. ALP response was defined as a reduction from baseline of ≥30%, confirmed ≥4 weeks later [12]; ALP nonresponse was defined as a change of <30% or an increase of ≥25% in ALP from baseline. In an additional exploratory analysis among patients with elevated tALP_BL_, ALP response was defined as a reduction of ≥10% from baseline and ALP nonresponse was defined as a change of <10% or an increase from baseline.

OS was assessed in relation to ALP_BL_ comparing those with normal ALP_BL_ and those with elevated ALP_BL_, and comparing ALP responders (≥30% reduction from baseline or ≥10% reduction from baseline among patients with elevated tALP_BL_) and ALP nonresponders. ALP_max_ was the greatest percentage reduction in ALP from baseline in any treatment cycle, and was assessed in patients with elevated tALP_BL_ only (patients with normal ALP_BL_ were excluded because they would not be expected to show a further significant reduction). The correlation between ALP_max_ and OS (*r*^2^) was also determined.

#### Lymph node disease

The presence of coexisting bone and lymph node metastases was determined using cross-sectional imaging. Lymph node disease categories were as follows: *group 1* baseline lymph node disease, *group 1a* lymph node disease progression during treatment, *group 2* no evidence of lymph node disease at any stage, and *group 3* development of new lymph node disease during treatment. For each lymph node disease category, OS and EOBD category were determined.

### Statistical methods

Analyses were performed using Excel and Statsdirect. OS was analysed using Kaplan-Meier survival curves. Peto’s log-rank test was used to determine whether the survival curves were significantly different between groups.

## Results

### Patient population

In year 1 (March 2014–2015), 26 patients were included, and in year 2 (March 2015–2016), 31 patients were included. Baseline characteristics of the patients are shown in Table 1. Overall, 14 of the 26 patients (54%) completed five or six cycles in year 1, and 17 of the 31 patients (55%) completed five or six cycles in year 2. Median follow-up was 298 days.

**Table 1:** Baseline characteristics of the 57 patients included in this study and the 614 patients included in the ^223^Ra-dichloride group of the ALSYMPCA trial [12]

Characteristic	This study	ALSYMPCA trial
Age (years), median (range)	74 (48–90)	71 (49–90)
Age >75 years, *n* (%)	23 (40)	171 (28)
Any prior use of docetaxel, *n* (%)		
Yes	32 (56)	352 (57)
No	25 (44)	262 (43)
Total alkaline phosphatase level (U/L), *n* (%)		
<220	28 (54)	348 (57)
≥220	24 (46)	266 (43)
0–130	16 (28)	NR
130–260	15 (26)	NR
260–1,000	21 (3)	NR
>1,000	4 (7)	NR
Eastern Cooperative Oncology Group performance status, *n* (%)		
0	1 (2)	165 (27)
1	34 (60)	371 (60)
≥2	22 (38)	77 (13)
Extent of bone disease (EOBD category), *n* (%)		
<6 metastases (EOBD1)	10 (18)	100 (16)
6–20 metastases (EOBD2)	16 (28)	262 (43)
>20 metastases (EOBD3)	17 (30)	195 (32)
Superscan (EOBD4)	14 (25)	54 (9)
Baseline blood investigations, median (range)		
Haemoglobin (g/dL)	12.1 (7.6–14.6)	12.2 (8.5–15.7)
Total alkaline phosphatase (U/L)	240 (58–2,805)	211 (32–6,431)
Prostate-specific antigen (μg/L)	223.9 (0.61–1,747)	146 (4–6,026)
Other treatments, *n* (%)		
Adjuvant therapy during ^223^Ra-dichloride	50 (88)	NR
Antiandrogen	40 (70)	NR
Steroids	34 (60)	NR
External beam radiation therapy	12 (21)	99 (16)^a^
Abiraterone	7 (12)	NR
Enzalutamide	7 (12)	NR
Bisphosphonates	NR	250 (41)^b^

*NR* not reported

^a^Within 12 weeks of therapy

^b^''Current use'

### Referral patterns

**Fig. 1:**
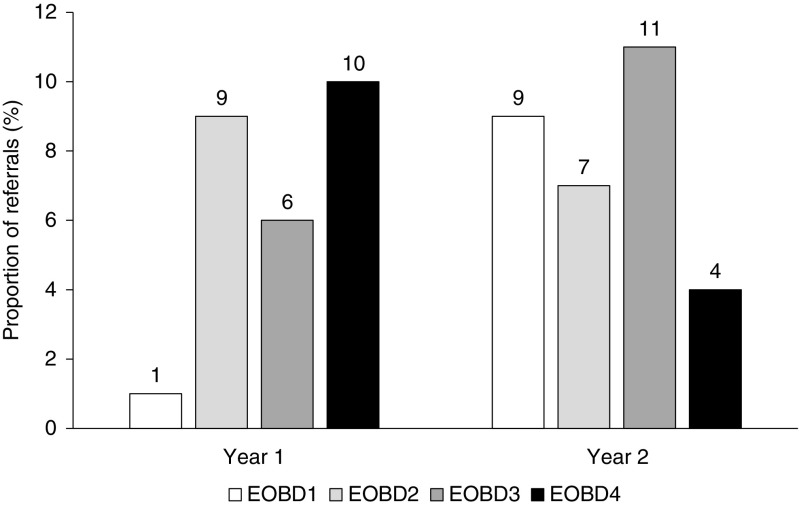
Pattern of referrals from uro-oncology to nuclear medicine in year 1 and year 2, in relation to the extent of bone disease (EOBD) category among the 57 included patients

The proportions of patients with EOBD1 referred to the Nuclear Medicine Department were 4% ( 1/26 patients) in year 1 and 29% (9/31 patients) in year 2, an increase of 25%. The proportions of patients with EOBD4 referred were 38% (10/26 patients) in year 1 and 13% (4/31 patients) in year 2, a decrease of 25%. Overall, referral rates of patients with EOBD2 and EOBD3 remained largely similar (Fig. 1).

### Effect of referral patterns on outcomes

**Fig. 2:**
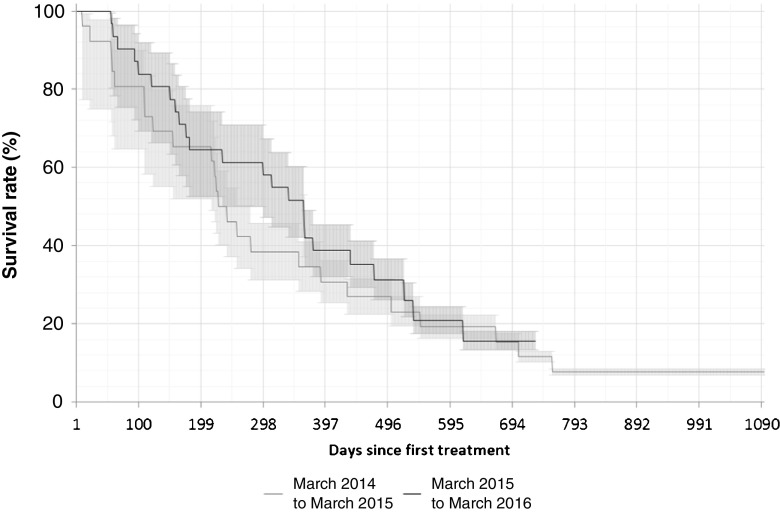
Overall survival in 26 patients in year 1 and 31 patients in year 2. Median survival times: 233 days in the total population, 227 days in year-1 patients, and 266 days in year-2 patients. Median follow-up times: 298 days in the total population, 234 days in year-1 patients, and 363 days in year-2 patients (*p* = 0.55, year 1 vs. year 2)

In the total population, OS in year 2 was longer than in year 1, but the difference was not statistically significant (227 vs. 266 days, *p* = 0.55; Fig. 2). In parallel with a lower bone disease burden, the incidence of SSREs was lower in year 2 than in year 1 (9.6% and 19.2%, respectively; Table 2).

**Table 2:** Incidence of symptomatic skeleton-related events in year 1 and year 2 in relation to extent of bone disease (EOBD) category

Event	EOBD category
Year 1 (5/26 [19.2%])	
External radiation therapy for pain (spine)	2
Hip fracture	3
MSCC and skull metastases with symptoms	3
MSCC	4
MSCC and femoral fracture	4
Year 2 (3/31 [9.6%])	
Lumbar spine pain causing admission	2
MSCC	3
External radiation therapy for pain (spine and pelvis)	3

*MSCC* metastatic spinal cord compression

### Prognostic factors

#### EOBD category

**Fig. 3:**
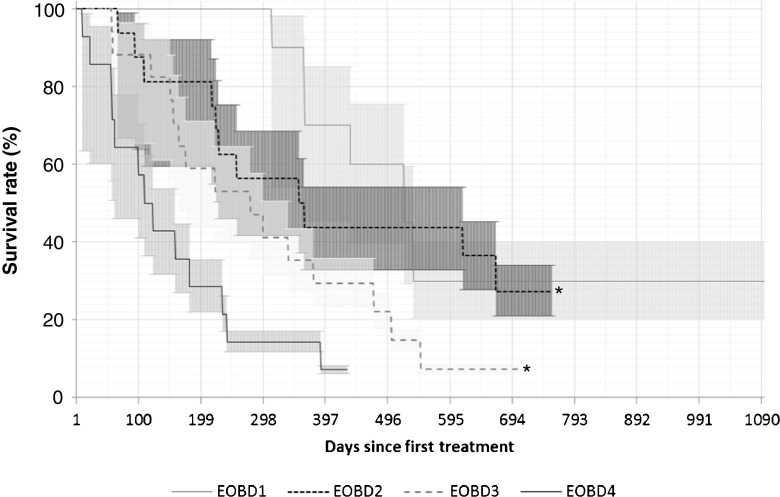
Overall survival in relation to extent of bone disease (EOBD) category: 10 patients with EOBD1, 16 with EOBD2, 17 with EOBD3 and 14 with EOBD4. Median survival times: 437 days, 242 days, 250 days, and 116 days in patients with EOBD1, EOBD2, EOBD3 and EOBD4, respectively. Median follow-up times: 485 days, 359 days, 278 days, and 116 days, respectively. *p < 0.05 vs. EOBD4

OS curves for patients with each EOBD category are shown in Fig. 3. The numbers of patients in each group who died were as follows: 7 (70%) with EOBD1, 12 (75%) with EOBD2, 16 (94%) with EOBD3 and 14 (100%) with EOBD4. There were significant differences in OS between patients with EOBD4 and all other groups (EOBD1 vs. EOBD4, *p* = 0.001; EOBD2 vs. EOBD4, *p* = 0.002; EOBD3 vs. EOBD4, *p* = 0.02), and between patients with EOBD1 and those with EOBD3 (*p* = 0.04). During both year 1 and year 2, the proportions of patients completing six cycles of treatment were far higher in patients with EOBD1 (100% and 89%, respectively) than in those with EOBD4 (20% and 25%, respectively; Table 3).

**Table 3:** Number (%) of patients completing ^223^Ra-dichloride treatment cycles in year 1 and year 2, in relation to extent of bone disease (EOBD) category

	Number of treatment cycles						Total
	1	2	3	4	5	6	
Year 1							
EOBD1						1 (100)	1
EOBD2			3 (33)	1 (11)	1 (11)	4 (44)	9
EOBD3			1 (17)		1 (17)	4 (67)	6
EOBD4	4 (40)	2 (20)	1 (10)		1 (10)	2 (20)	10
Total	4 (15)	2 (8)	5 (19)	1 (4)	3 (12)	11 (42)	26
Year 2							
EOBD1				1 (11)		8 (89)	9
EOBD2		1 (14)	1 (14)		2 (29)	3 (43)	7
EOBD3	2 (18)	2 (18)	2 (18)	2 (18)		3 (27)	11
EOBD4	1 (25)			2 (50)		1 (25)	4
Total	3 (10)	3 (10)	3 (10)	5 (16)	2 (6)	15 (48)	31

#### Alkaline phosphatase

**Fig. 4:**
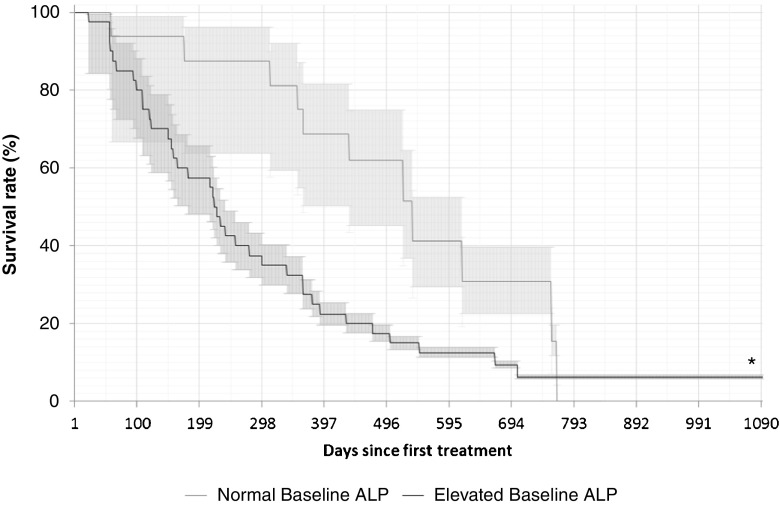
Overall survival in relation baseline alkaline phosphatase (ALP_BL_) status (normal in 16 patients, elevated in 40; one patient did not have any baseline data). Median survival times: 401 days in patients with normal ALP_BL_, and 222 days in those with elevated ALP_BL_. Median follow-up times: 468 days in patients with normal ALP_BL_, and 234 days in those with elevated ALP_BL._ ,. *p = 0.01 vs. normal ALP_BL_

**Fig. 5:**
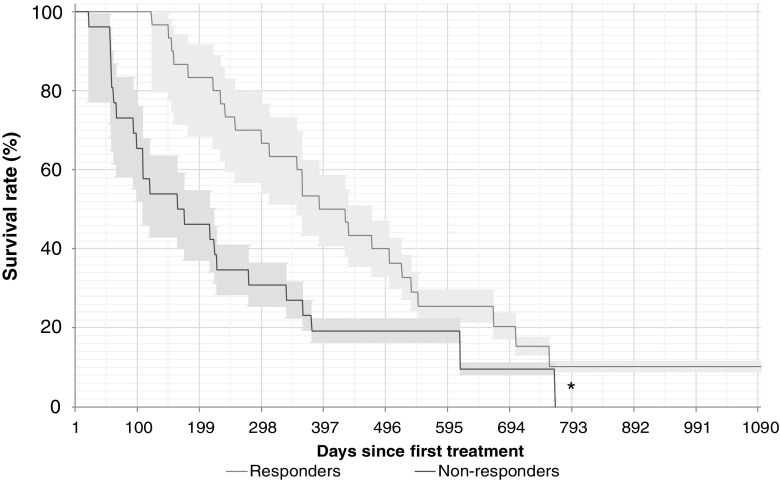
Overall survival in relation to alkaline phosphatase (ALP) response (≥30% vs. <30% reduction from baseline; 30 responders, 26 nonresponders). Median survival times: 363 days in ALP responders, and 115 days in ALP nonresponders. Median follow-up times: 411 days in ALP responders, and 170 days in ALP nonresponders. *p = 0.01 vs. ALP responders

**Fig. 6:**
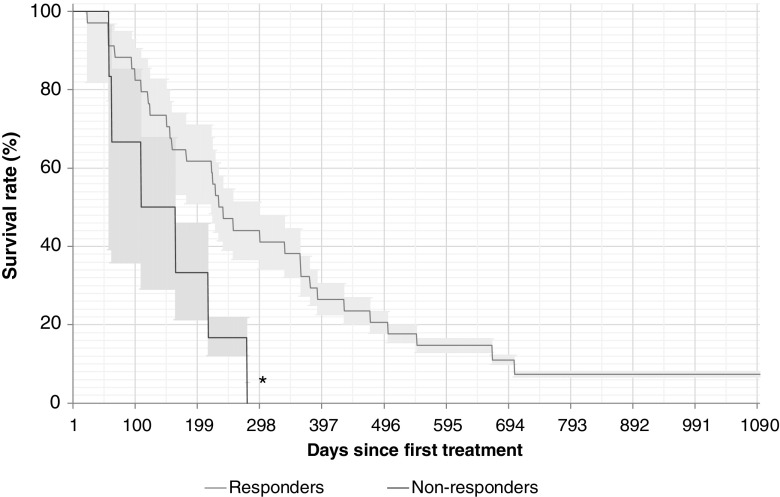
Overall survival in relation to alkaline phosphatase (ALP) response among patients with elevated total ALP at baseline (≥10% vs. <10% reduction from baseline; 34 responders, 6 nonresponders). Median survival times: 256 days in ALP responders, and 137 days in ALP nonresponders. Median follow-up times: 325 days in ALP responders, and 216 days in ALP nonresponders. *p = 0.03 vs. ALP responders

**Fig. 7:**
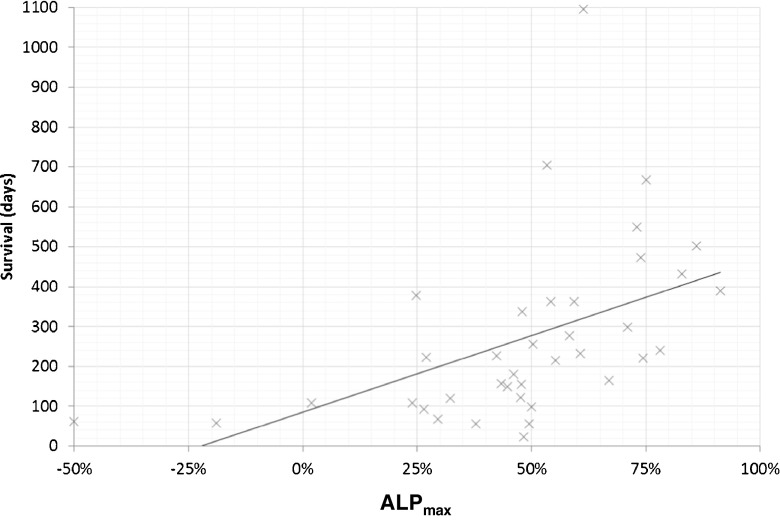
Correlation between overall survival and greatest percentage reduction in total alkaline phosphatase (tALP) from baseline in any treatment cycle (ALP_max_) among 40 patients with elevated tALP at baseline (*r*^2^ = 0.24)

OS was significantly longer among patients with normal ALP_BL_ than among those with elevated ALP_BL_ (401 vs. 222 days, *p* = 0.01; Fig. 4), and among ALP responders (≥30% reduction) than among ALP nonresponders (363 vs. 115 days, *p* = 0.01; Fig. 5). When ALP response was defined as a reduction of ≥10% from baseline (exploratory analysis among patients with elevated ALP_BL_ only), OS was significantly longer among ALP responders than among ALP nonresponders (256 vs. 137 days, *p* = 0.03; Fig. 6). Among patients with elevated ALP_BL_, a correlation was also observed between OS (233 days) and ALP_max_ (*r*^2^ = 0.24; Fig. 7). The correlation between OS and ALP_max_ was weaker among patients with normal ALP_BL_ (*r*^2^ = 0.17).

#### Lymph node disease

Recent cross-sectional imaging data (CT and/or magnetic resonance imaging, MRI) were available in 46 patients who were included in the analysis of lymph node disease. Over a quarter of the patients had baseline lymph node disease (group 1; 13/46 patients, 28%). Of these, six patients progressed during treatment (group 1a; 6/13, 46%). There was no evidence of lymph node disease at baseline or during treatment in 30 patients (group 2; 30/46, 65%). New lymph node disease developed in three patients (group 3; 3/46, 7%).

**Fig. 8:**
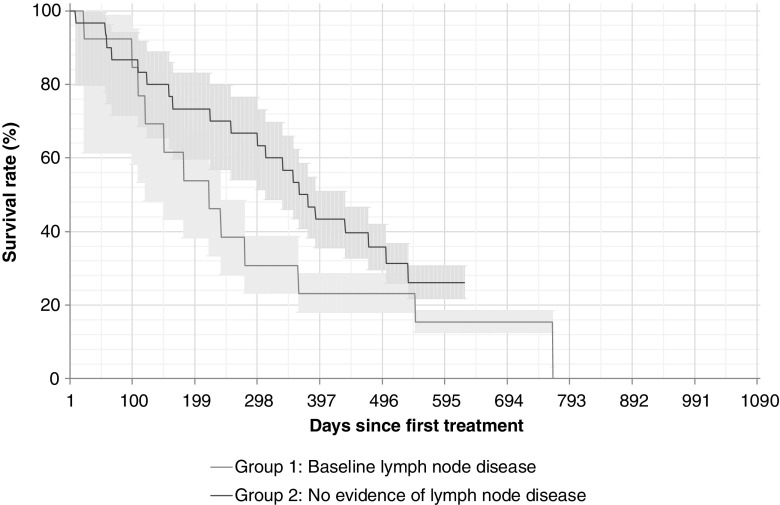
Overall survival in relation to the presence lymph node disease (13 patients with lymph node disease, 30 patients without). Median survival times: 181 days in those with lymph node disease, and 325 days in those without lymphadenopathy at any stage. Median follow-up times: 221 days in those with lymph node disease, and 371 days in those without lymphadenopathy at any stage. *p* = 0.29 for presence vs. absence of lymph node disease

The presence of coexisting lymph node disease appeared to correlate with bone disease burden: in lymph node disease group 1, more patients had EOBD3 (5/13 patients, 38%) or EOBD4 (4/13, 31%) than EOBD1 (2/13, 15%) or EOBD2 (2/13, 15%). The same was true of lymph node disease group 1a. OS was shorter among patients with coexisting lymph node metastasis at baseline or with lymph node progression than in those without lymphadenopathy at any stage (181 days vs. 325 days, *p* = 0.29; Fig. 8).

## Discussion

Prior to this study, patients were often referred to the Nuclear Medicine Department at this centre with very late-stage disease and severe metastatic bone burden; indeed, many clinicians were waiting for ^223^Ra-dichloride clinical approval (which was granted on the basis of the ALSYMPCA trial findings) before referring patients. At baseline, the prevalence of diffuse bone metastases (so-called ‘superscan’) at referral in the overall population during both year 1 and year 2 was 25% (10 of 26 patients, 38%, in year 1; 4 of 31 patients, 13%, in year 2; i.e. 14 of 57 patients, 25%, in total), compared with 9% in the ALSYMPCA trial [12]. These patients with a superscan would often not complete the treatment course. Our study showed that the pattern of referrals reversed from year 1 to year 2 of ^223^Ra-dichloride service, with a marked increase in referrals of patients with EOBD1 and a decrease in referrals of those with EOBD4. The pattern of referrals in patients with EOBD2/3 remained similar between year 1 and year 2. The implementation of a strong collaborative uro-oncology/nuclear medicine multidisciplinary referral pathway led to referring clinicians having increased confidence in earlier intervention and greater success of treatment with ^223^Ra-dichloride.

In this analysis, EOBD (as determined by bone scintigraphy) was shown to be a predictor of OS in patients treated with ^223^Ra-dichloride. Treating more patients with a lower bone burden resulted in improved overall outcomes, including a reduction in the incidence of SSREs and an increase in OS. The rates of SSREs observed in this study (19.2% in year 1, 9.6% in year 2) were much lower than that found in the ALSYMPCA trial (35%), in spite of the fact that the studies used the same definition of SSRE. This may be due to a longer follow-up in the ALSYMPCA trial and/or further advances in CRPC treatment since the ALSYMPCA trial and overall better bone health in the more recently recruited patients, but this interesting aspect requires further investigation in prospective studies. We also expect that SSREs may increase over time due to the natural course of the disease. In our sample, SSREs were observed in patients with EOBD2, 3 and 4 in year 1, and in patients with EOBD2 and 3 in year 2.

EOBD was also shown to be predictive of survival in patients receiving ^223^Ra-dichloride treatment in the ALSYMPCA trial. The median OS, however, was shorter in our study (233 days) than in the ALSYMPCA trial (14.9 months). This may have been due to the higher burden of disease in our patients than in the ALSYMPCA trial patients: our patients included a higher proportion of with a superscan (overall 25% for both years vs. 9%), a much lower proportion with EOBD2 (28% vs. 43%), a higher proportion with ECOG performance status ≥2 (38% vs.13%) and a lower proportion with ECOG performance status 0 (2% vs. 27%), and the median baseline PSA level was higher in our patients (223.9 vs. 146 μg/L; Table 1). It may also have been a result of differences in the definition of OS: in our study, OS was defined from the first dose (i.e. first visit to the nuclear medicine clinic), whereas in the ALSYMPCA trial OS was defined from randomization, which may or may not have been when the first ^223^Ra-dichloride dose was administered [12]. A small case series (15 patients) showed differing results, indicating that baseline EOBD is not indicative of response to ^223^Ra-dichloride therapy. However, the authors did note a trend towards an unfavourable response to treatment among those presenting with EOBD4 [16].

In our study, tALP was useful as a predictor of OS in patients receiving ^223^Ra-dichloride. ALP responders had significantly longer OS than ALP nonresponders (363 vs. 115 days for a reduction of ≥30% from baseline and 256 vs. 137 days for a reduction of ≥10% from baseline among patients with elevated ALP_BL_; *p* < 0.05 for both). In addition, among patients with elevated ALP_BL_, those with the greatest reductions in ALP in any treatment cycle (ALP_max_) generally had a longer OS. We have previously hypothesized that tALP dynamics and ALP_max_ are more relevant in patients with elevated tALP_BL_ than in those with normal ALP_BL_ [17]. In the present study, patients with normal tALP_BL_ had a longer OS than those with elevated tALP_BL_ (401 vs. 222 days; *p* = 0.01). Changes in tALP were less clear in those with normal tALP_BL_, as demonstrated by the fact that the correlation between OS and ALP_max_ was stronger among patients with elevated ALP_BL_ than normal ALP_BL_ (*r*^2^ = 0.24 and *r*^2^ = 0.17, respectively). Similar trends were observed in the ALSYMPCA trial: in patients with elevated tALP_BL_ (defined as ≥220 U/L), ^223^Ra-dichloride treatment was associated with longer OS than placebo treatment. In patients with tALP_BL_ <220 U/L, there was a trend towards a longer OS with ^223^Ra-dichloride than with placebo, but the confidence intervals crossed the line of unity [12].

In a recent study, a significant reduction in tALP occurred as early as 4 weeks after initiating ^223^Ra-dichloride therapy [18]. Reductions in tALP in week 12 correlated with longer OS, but did not meet statistical requirements for use as a surrogate marker of survival. The authors suggested, therefore, that dynamic changes in tALP may be useful for monitoring but not as a surrogate marker. It is of note, however, that dynamic changes were analysed in all patients, including those with normal baseline tALP – in contrast to our study in which changes were analysed only in patients with elevated tALP_BL_. The utility of tALP alone is somewhat limited by the fact that it may increase for reasons other than treatment failure, for example the development of liver metastases [19]. However, ALP response has also been shown to be associated with survival in patients receiving chemotherapy [18, 20]. In the TAX327 trial among men with high baseline ALP receiving docetaxel or mitoxantrone, normalization of ALP was associated with longer OS, while an increase in ALP was associated with poor OS. This was independent of changes in PSA levels [21].

Regarding co-existent lymph node and bone metastases and survival, the site of metastases may have prognostic implications. Patients with bone metastases have shorter survival than patients with lymph node metastases only, and mortality has been shown to increase as the disease progresses from lymph nodes to bone to visceral tissue [22]. However, prostate cancer often metastasizes to bone first, with lymph node disease developing later. Our data suggest that coexistent lymph node disease or lymph node disease progression during ^223^Ra-dichloride treatment may be predictive of a shorter OS. Lymph node disease appears to be associated with higher bone disease burden, but may be present even in patients with EOBD1 or EOBD2. Patients with coexisting bone and lymph node metastases should therefore be considered for combined ^223^Ra-dichloride treatment with, for example, chemotherapy or beta-emitting ^177^Lu-PSMA-617 at an earlier stage. Large prospective studies are required to further assess the effect of ^223^Ra-dichloride on outcomes in this patient group, and to determine whether coexisting lymph node disease may be an indicator of survival [23].

In the ENTHUSE M0 trial, more than 30% of patients whose CRPC was thought to be nonmetastatic (M0) in fact had metastatic disease when assessed by MRI, CT or bone scan. This unexpectedly high rate of metastatic disease suggests that regular imaging should be considered in CRPC, even in the absence of symptoms [24]. In one of our previous studies, cross-sectional imaging within 3 months of initiating ^223^Ra-dichloride therapy improved completion rates. This is an issue that needs to be addressed in treatment guidelines [25].

There were several limitations in this analysis. While OS was, to a certain degree, longer in year 2 than in year 1, the difference was not statistically significant, and this may have been due to the relatively small sample size. The proportion of patients with EOBD2 was far lower than that in the ALSYMPCA trial (28% vs. 43% [12]) and was slightly lower in year 2 than in year 1; this too may have had an inverse impact on the OS. Furthermore, to receive ^223^Ra-dichloride, patients were required to be castration-resistant with symptomatic bone metastases, irrespective of when they had been labelled as castration-resistant. As a result, in some patients the time since becoming castration-resistant was longer, and hence these patients were likely to have had a higher metastatic burden and poorer outcomes. It is also not clear whether patients were truly being referred earlier (i.e. whether patients with earlier stage disease, who may not previously have been considered eligible for referral, were being referred in year 2) or whether patients with later stage disease (i.e. superscan patients) were no longer being referred.

This study has helped identify a number of opportunities for further research. These include the following:

- Selection of patients for retreatment with ^223^Ra-dichloride.
- The impact of intervention at earlier stages of bone disease.
- The use of ^223^Ra-dichloride in asymptomatic patients (not currently on-label).
- The efficacy of ^223^Ra-dichloride and abiraterone and/or enzalutamide. Note that the combination of ^223^Ra-dichloride, abiraterone and prednisone/prednisolone is currently contraindicated based on evidence suggesting increased mortality and fracture risk with this combination in the ERA 223 trial in patients with mCRPC, bone metastases and mild or no symptoms. It is important to note that the safety and efficacy of ^223^Ra-dichloride in combination with second-generation androgen receptor antagonists, such as enzalutamide, have not yet been established; however, to date, no safety issues have been reported [26].
- The efficacy of an as-yet-unexplored combination or ‘cocktail’ of alpha-emitting radium and beta-emitting ^177^Lu-PSMA radioligand therapy (due to a better response of lymph node metastases than bone metastases [27], but transient side effects on the salivary and lacrimal glands in contrast to alpha-emitting PSMA treatment, but also due to a possible synergistic effect of the two different types of emission).
- The identification of the best imaging and biochemical markers for assessing response to ^223^Ra-dichloride.

### Conclusion

In this study, the increased confidence of referring clinicians following the introduction of a strong collaborative multidisciplinary referral pathway led to earlier intervention and improved outcomes in patients treated with ^223^Ra-dichloride. Monitoring dynamic changes in tALP is more relevant in patients with elevated tALP_BL_ than in those with normal tALP_BL_; patients with normal tALP_BL_ had a longer OS than those with elevated tALP_BL_, but changes in tALP were less clear in those with normal tALP_BL_. Imaging and prognostic markers may be of value for individualizing ^223^Ra-dichloride treatment and planning retreatment, and their integration into treatment guidelines should be considered.
